# Primary ovarian insufficiency and myasthenia gravis

**DOI:** 10.12669/pjms.36.3.2124

**Published:** 2020

**Authors:** Saleh A. AlAsiri

**Affiliations:** 1 Saleh A. AlAsiri, MD, FRCSC, FACOG, FACS, FICS. Assistant Professor and Consultant, Reproductive Endocrinology and infertility, In vitro Fertilization Unit, Department of Obstetrics and Gynecology, College of Medicine, King Saud University & King Saud University Medical City (KSUMC), Riyadh. PO Box 7805, Riyadh 11472, Saudi Arabia

**Keywords:** Primary Ovarian Insufficiency, Myasthenia Gravis

## Abstract

Myasthenia gravis (MG) is an autoimmune disease. In some patients, Primary Ovarian Insufficiency (POI) is considered to be an autoimmune condition. Their coexistence is rare and suggests autoimmune perturbation. We describe a patient who was determined to have POI at 17 years of age and who developed MG 20 years later.

## CASE REPORT

A 37-year-old nulligravid woman with drooping of the right eyelid and double vision, easy fatiguability and weakness in the upper limbs. After the diagnosis of Myasthenia Gravis (MG) was confirmed by positive neostigmine test, she was put on oral pyridostigmine, 60 mg three times a day. She denied any association of MG symptoms with menstruation. Her past medical history was remarkable for delayed puberty and primary amenorrhea.

Hypergonadotrophic hypogonadism had been diagnosed when she was 17 years old, and hormone replacement therapy had been instituted. Her family history was noncontributory. On physical examination, height was 175 cm, weight 58 kg with a BMI of 18.9. Her breasts were small, at Tanner stage III. Musculoskeletal system and neurologic examination disclosed a motor power of 5/5 before the fatigability test that decreased to 4/5 after the test. Her quantitative MG score was 35/39. Karyotype revealed normal 46XX status. On pelvic imaging, patient had small uterus with “streak” ovaries consisting of sparse stroma characteristically devoid of follicles. Computed tomography of the mediastinum showed a slightly enlarged thymus. The patient was ambivalent about undergoing thymectomy.

## DISCUSSION

Primary Ovarian Insufficiency (POI) in association with MG has been reported only rarely,^1^,^2^ suggesting autoimmunity as a common etiopathological process involved in these disorders. POI and MG are believed to coexist in less than 1% of patients.^1^ It has been reported that MG immunotherapy may restore fertility in affected women.^2^

Despite the presence of evidence for autoimmunity in the form of positive anti-nuclear antibody test and anti-thyroid antibodies, circulating anti-ovarian antibodies were not detected in the patient in this case. The significance of the presence of circulating anti-ovarian antibodies, despite their low prevalence (16.67%) in patients with ovarian pathology, remains controversial. The presence of anti-ovarian antibodies does not imply their etiopathological role (and may be a transient, short-lived phenomenon), and their predictive value in identifying autoimmune ovarian failure is poor. In our patient, the absence of circulating anti-ovarian antibodies could have been due to the interval of almost 20 years between the diagnosis of the two disorders or to the lack of antigenic stimulation resulting from autoimmune loss of ovarian tissues.

**Table-I:** Autoantibody profile of the patient.

Antibody	Result	Normal Range
Anti-thyroid peroxidase antibodies	Positive	
Anti-thyroglobulin antibodies (anti-Tg)	85 IU/mL	0–60 IU/mL
Anti-microsomal antibodies (anti-M)	600 IU/mL	0–100 IU/mL
Celiac antibody profile	Negative	
Rheumatoid factor	<10 IU/mL	
Antinuclear antibody	1:160	<1:40
Double stranded DNA antibodies	Negative	
Anti cardiolipin antibodies IgM and IgG	Negative	
Anti-acetylcholine receptor binding antibodies	> 80 nmol/L	Negative: 0.00–0.24 Borderline: 0.25–0.40 Positive: > 0.40
Anti-acetylcholine receptor blocking antibodies	51%	Negative: 0–25% Borderline: 26–30% Positive: >30%
Anti-acetylcholine receptor modulating antibodies	45%	0–20%
Anti-adrenal antibodies	Negative	
Anti 21-hydroxylase antibodies	< 1 U/mL	< 1 U/mL
Anti-ovarian antibodies	Negative	

Autoimmune mechanism is considered to be a cause of POI in about 20–30% of patients. In up to 50% of patients with autoimmune POI, there is evidence for the presence of other autoimmune disorders.^3^

The most frequently coexisting autoimmune disorder may be autoimmune thyroid disease, which is found in up to 33% of patients with POI.^4^ Spontaneous pregnancy after correction of hypothyroid status has been reported in a patient who had both ovarian and thyroid failure for 11 years, indicating an association between the two conditions. A strong association between autoimmune POI and autoimmune adrenal insufficiency (Addison’s disease) has been reported. The patient in this case had circulating thyroid autoantibodies, but adrenal antibodies were not detected. It is, however, interesting to note that antinuclear antibodies were present in high titer with undetectable extractable nuclear antigens, and the patient had a normal 46 XX karyotype. Patients with POI with abnormal karyotypes tend not to harbor antinuclear antibodies as frequently as the POI patients with normal karyotype, particularly when they are less than 30 years of age.^5^ These POI patients with normal karyotype and positive reactivity to antinuclear antibody have been classified both etiologically and clinically as a distinct group.

This case report highlights a rare association of POI and MG. Physicians should be vigilant in their assessment of patients presenting with either of these conditions to ensure their clinical management and counseling are appropriate and effective. As the interval between the diagnosis of POI and MG can be very long, close follow-up is strongly recommended.
